# Phytochemical and Biological Screening of *Oenothera biennis* L. Hydroalcoholic Extract

**DOI:** 10.3390/biom10060818

**Published:** 2020-05-26

**Authors:** Ramona Fecker, Valentina Buda, Ersilia Alexa, Stefana Avram, Ioana Zinuca Pavel, Delia Muntean, Ileana Cocan, Claudia Watz, Daliana Minda, Cristina Adriana Dehelean, Codruta Soica, Corina Danciu

**Affiliations:** 1 Department of Pharmacognosy, University of Medicine and Pharmacy “Victor Babeş”, Eftimie Murgu Square, No. 2, 300041 Timişoara, Romania; mioritic2000@gmail.com (R.F.); stefana.avram@umft.ro (S.A.); ioanaz.pavel@umft.ro (I.Z.P.); daliana.minda@umft.ro (D.M.); corina.danciu@umft.ro (C.D.); 2 Department of Pharmacology and Clinical Pharmacy, University of Medicine and Pharmacy “Victor Babeş”, Eftimie Murgu Square, No. 2, 300041 Timişoara, Romania; 3 Department of Food Control, Banat’s University of Agricultural Sciences and Veterinary Medicine “King Michael I of Romania” from Timişoara, Calea Aradului No. 119, 300641 Timişoara, Romania; alexa.ersilia@yahoo.ro (E.A.); negreaileana@yahoo.com (I.C.); 4 Department of Microbiology, University of Medicine and Pharmacy “Victor Babeş”, Eftimie Murgu Square, No. 2, 300041 Timişoara, Romania; 5 Department of Pharmaceutical Physics, University of Medicine and Pharmacy “Victor Babeş”, Eftimie Murgu Square, No. 2, 300041 Timişoara, Romania; farcas.claudia@umft.ro; 6 Department of Toxicology, University of Medicine and Pharmacy “Victor Babeş”, Eftimie Murgu Square, No. 2, 300041 Timişoara, Romania; cadehelean@umft.ro; 7 Department of Pharmaceutical Chemistry, University of Medicine and Pharmacy “Victor Babeş”, Eftimie Murgu Square, No. 2, 300041 Timişoara, Romania; codrutasoica@umft.ro

**Keywords:** *Oenothera biennis* L. hydroalcoholic extract, antibacterial, antiproliferative, pro-apoptotic, mitochondrial function, chorioallantoic membrane, angiogenesis, inflammation

## Abstract

*Oenothera biennis* L. (OB), also commonly known as evening primrose, belongs to the Onagraceae family and has the best studied biological activity of all the members in the family. In therapy, the most frequently used type of extracts are from the aerial part, which are the fatty oils obtained from the seeds and have a wide range of medicinal properties. The aim of this study was to evaluate the phytochemical composition and biological activity of OB hydroalcoholic extract and to provide directions for the antimicrobial effect, antiproliferative and pro-apoptotic potential against A375 melanoma cell line, and anti-angiogenic and anti-inflammatory capacity. The main polyphenols and flavonoids identified were gallic acid, caffeic acid, epicatechin, coumaric acid, ferulic acid, rutin and rosmarinic acid. The total phenolic content was 631.496 µgGAE/mL of extract and the antioxidant activity was 7258.67 μmolTrolox/g of extract. The tested extract had a mild bacteriostatic effect on the tested bacterial strains. It was bactericidal only against *Candida* spp. and *S. aureus*. In the set of experimental conditions, the OB extract only manifested significant antiproliferative and pro-apoptotic activity against the A375 human melanoma cell line at the highest tested concentration, namely 60 μg/mL. The migration potential of A375 cells was hampered by the OB extract in a concentration-dependent manner. Furthermore, at the highest tested concentration, the OB extract altered the mitochondrial function in vitro, while reducing the angiogenic reaction, hindering compact tumor formation in the chorioallantoic membrane assay. Moreover, the OB extract elicited an anti-inflammatory effect on the experimental animal model of ear inflammation.

## 1. Introduction

Phytotherapy has regained huge interest and confidence in the last two to three decades, although it was present and used in all human cultures for centuries. It has become a mainstream therapy in many countries (developed or in developing), and is frequently used as a primary source of healthcare because of its well-known advantages, including its efficiency in a wide range of pathologies and milder side effects, as compared to chemical-synthetized molecules. Moreover, the enormous costs of finding and developing modern drugs and the high price of the currently available drugs contributed to the need to look for and reuse herbal supplements and phytonutrients. However, it is unanimously accepted that phytotherapy has its limitations. Scientists today have made significant strides in order to obtain better knowledge and understanding about the properties, the therapeutic indications and the risks associated with this approach (with promising potential and efficacy in several pathologies) [1,2,3,4].

*Oenothera biennis* L. (OB), also known as evening primrose, belongs to the Onagraceae family. It is the most predominant and studied species of its family. With research conducted for more than 120 years on the entire family, the first reports of this plant are associated with the early development of genetics [5,6]. Originating in America (temperate and tropical climate zones), the *Oenothera* genus (the second largest genus of flowering plants) is currently present in various zones of the planet and include approximately 145 species, which are divided into 18 sections, from which 70 species can be found in Europe [5,6]. Evening primrose has the best studied biological activity of all the members of the family and possess a wide range of medicinal properties [6].

In therapy, the most frequently used types of extracts are from the aerial part, which are, respectively, the fatty oils obtained from the seeds. From a chemical point of view, in the aerial part of the plant, the representative compounds are a part of the class of flavonoid glycosides (kaempferol-3-O-glucoside, quercetin-3-O-galactoside, quercetin-3-O-rhamnoside, myricetin-3-O-glucoside), phenolic acids (ellagic, gallic, caffeic, coumaric), and tanins. In seeds, there is a concentration of about 24% fatty oil, consisting mainly of linolic, γ-linolenic, oleic, palmitic, and stearic acids. The seeds also contain proteins (15%), lignans and cellulose (43%). The amount of the oil that can be found in the seeds depends on the age of the seeds, cultivation and growth conditions. Moreover, in the aqueous extract of the leaves, phenolic compounds (ellagitannins and caffeoyl tartaric acid), flavonoids (quercetin glucuronide; kaempferol glucuronide), carbohydrates (arabinose, glucose, galactose, mannose, glucuronic and galacturonic acid) and tannins (oenothein A; oenothein B) can be detected. Timoszuk et al. also described the phytochemical composition of methanolic extracts obtained from the root of evening primrose. They identified sterols (sitosterol, oenothera lanosterol A; oenothera lanosterol), pentacyclic triterpene type maslinic acid and oleanolic acid, carbohydrates, tannins, xanthone and its derivates [5,7].

Due to an increased number of active phytochemicals found in several areas of the plant (aerial parts, leaf, roots, and seeds) and the fact that the oil extracted from evening primrose represents an important source of gamma-linoleic acid (GLA) and linoleic acid (LA), it possess several important pharmacological properties, such as (a) anti-oxidant (all the plant extracts or oil-triterpenoid esters were found to be effective in reducing oxidative stress and have radical scavenging activity) [8,9]; (b) anti-inflammatory (extracts from aerial parts of the oenotherin B can inhibit lipoxygenase and hyaluronidase in a concentration-dependent dose and the sterols found in the oil have a protective effect on some inflammatory mediators) [8,10]; (c) anti-diabetic (the extract has an important role in decreasing postprandial hyperglycemia, while the oil associated with vitamin D has been shown to improve the glycemia and lipid profile in women with gestational diabetes, with no effect on high-density lipoprotein (HDL)-cholesterol) [11,12]; (d) anti-cancer and anti-tumor (extracts/phytocompounds obtained from roots or seeds have shown antiproliferative, antiangiogenic, antimigratory, and antimetastatic effects against prostate, breast, hepatic cancer and leukemia cell lines) [13,14]; (e) anti-bacterial (extracts obtained from seed have been shown to have important actions against *Pseudomonas aeruginosa*, *Staphylococcus aureus*, *Escherichia coli*, and *Candida albicans*) [15]; (f) anti-neuropathic (GLA and LA concentrations have been shown to improve nerve function and symptoms in patients suffering from induced chemotherapy neuropathy) [16]; (g) hypolipemiant (GLA and LA have the ability to lower the increased total serum cholesterol and triglycerides concentration) [17]; (h) anti-thrombotic (the oil represents a source of prostaglandin E1—ameliorating the endothelial function) [18,19]; (i) cariostatic (seed extract has been shown to have inhibitory effects on dental caries in rats) [20]; (j) anti-ulcerogenic, cytoprotective and anti-helicobacter on several gastric lesions (seeds oil/extract) [21,22]; (k) anti-fungal (roots–gallic acid) [23]; (l) anti-acetylcholinesterase activity (aerial part extract) [24]; and (m) anti-viral (topical administration of the oil has been shown to represent a therapeutic alternative for children with *Molloscum contagiosum*) [25].

Summarizing all the information presented above, different parts of evening primrose, materialized in various types of extracts or isolated from pure active phytocompounds, can represent a therapeutic strategy for the following pathologies: atopic dermatitis, hyperlipidemia, atherosclerosis, endothelial dysfunction, cancer management, peptic ulcer, ulcerative colitis, Crohn’s disease, inflammatory bowel disease, multiple sclerosis, rheumatoid arthritis, children with *Molloscum contagiosum*, diabetes mellitus, bacterial and fungal infections, neurodegenerative disorders, and pre-menstrual syndrome [26,27,28,29,30]. All these aspects suggest the importance of this medicinal plant for therapy and represents real evidence that motivates further investigations as the chemical composition provides important active phytochemicals.

As a special observation, it is worth noting that precaution is needed when associating evening primrose oral supplements with other drugs, especially with drugs with narrow therapeutic indexes, such as anticoagulants and antivirals, due to a higher risk of bleeding, as well as a higher risk of side effects (evening primrose supplements are considered to exert an inhibitory action on the CYP3A4 isoenzyme of cytochrome P450) [31,32].

The aim of this study was to evaluate the phytochemical composition and biological activity of the OB hydroalcoholic extract and to provide directions towards the antimicrobial effect, antiproliferative and pro-apoptotic potential against the A375 melanoma cell line, anti-angiogenic and anti-inflammatory capacity.

## 2. Materials and Methods

### 2.1. Plant Materials

Dried aerial parts of OB were brought into Romania from the southern part of Tunisia (Djerba) by a student of the Victor Babeş University of Medicine and Pharmacy. The plant was identified in the department of Pharmacognosy and was assigned the voucher specimen code OB 11/2018. Extraction was conducted, as previously described [33]. Dried aerial parts were ground. Ten grams of plant material were brought to a powdery state and mixed with 50 mL of 70% ethanol. The extraction was performed in an ultrasonic bath (FALC LBS2, Treviglio, Italy) under the following conditions: 30 min, 40 kHz and 50 °C. After sonication, the OB extract was filtered with the help of a vacuum pump and the solvent was evaporated until dry, with the help of a rotary evaporator (250 bar pressure, temperature 60 °C, and 150 rpm). The OB extract was stored at −2 °C until use.

### 2.2. Determination of Individual Polyphenols

Characterization of the phenolic profile were performed at the Interdisciplinary Research Platform belonging to Banat’s University of Agricultural Sciences and Veterinary Medicine “King Michael I of Romania”. The individual polyphenols were detected using a Shimadzu Chromatograph (Kyoto, Japan) equipped with SPD-10A ultraviolet (UV, wavelength was 280 nm and 340 nm). The column used was EC 150/2 NUCLEODUR C18 Gravity SB 150 × 2 mm × 5 μm, and the Gradient elution was: mobile phases A: water acidified with formic acid at pH-3, B: acetonitrile acidified with formic acid at pH-3. Chromatographic separation: 0.01–20 min 5% B, 20.01–50 min 5%–40% B, 5–55 min 40%–95% B, 55–60 min 95% B. The solvent flow rate was 0.2 mL/min at 20 °C. The calibration curves were performed in the range of 20–50 μg/mL. The results were expressed in mg·g^−1^. Experiments were performed in duplicate. All the reagents and solvents used were analytical grade chemicals and all standards were prepared in methanol (Merck, KGaA, Darmstadt, Germany). The compounds identification was done using external standards of individual polyphenols. The calibration curves were as follows: ferulic acid, *y* = 1.172 × 10^−6^*x* (r = 0.9999); rosmarinic acid, *y* = 1.018 × 10^−6^*x* (r = 0.9982); gallic acid, *y* = 8.470 × 10^−6^*x* (r = 0.9996); coumaric acid, *y* = 1.1566 × 10^−6^*x* (r = 0.9997); caffeic acid, *y* = 7.110 × 10^−6^*x* (r = 0.9990); protochatecuic acid, *y* = 8.036 × 10^−6^*x* (r = 0.9990); epicatechin, *y* = 3.881 × 10^−5^*x* (r = 0.9996); rutin, *y* = 1.813 × 10^−5^*x* (r = 0.9999), resveratrol, *y* = 6.388 × 10^−6^*x* (r = 0.9945); quercetin, *y* = 1.001 × 10^−5^*x* (r = 0.9992); and kaempherol, *y* = 3.273 × 10^−5^*x* (r = 0.9990).

### 2.3. Determination of Total Polyphenols Content (TP)

The extract (0.1 g) was dissolved in 1 mL methanol and ultrasonicated for 30 min using Aqua Wave 9381 Barnstead Lab Line (Thermo Fischer, Waltham, MA, USA). 0.5 mL of the alcoholic extract was treated with 1.25 mL of Folin–Ciocalteu (Merck KGaA, Darmstadt, Germany) reagent diluted 1:10 with water. After incubation for 5 min at room temperature, 1 mL of 60 g/L Na_2_CO_3_ (S.C. Chemical Company S.A., Bucuresti, Romania) was added and the sample was incubated at 50 °C for 30 min. The absorbance of the samples was measured at 750 nm using an ultraviolet–visible (UV-VIS) spectrophotometer (Analytic Jena Specord 205, Jena, Germany). The calibration curve was obtained using gallic acid (GA) (Sigma Aldrich Chemie, Madrid, Spain) as standard with a blank methanol control (Merck KGaA, Darmstadt, Germany). The regression equation was *y* = 1.92*x* + 0.10 and the coefficient of correlation was r = 0.9980. The results were expressed in the µgGAE/mL extract. All the experiments were performed in triplicate.

### 2.4. Determination of the Total Antioxidant Activity (TAC)

The method used for determining the antioxidant activity (TAC) was the CUPRAC method. The CUPRAC (cupric-reducing antioxidant capacity) method is an electron-transfer (ET) method based on the absorbance measurement at 450 nm of Cu(I)-neocuproine (Nc) chelate, formed as a result of the redox reaction of chain-breaking antioxidants with the CUPRAC reagent, Cu(II)-Nc. The method depends on the reduction of the cupric neocuproine complex to the cuprous neocuproine complex by a reductant at low pH [34,35]. As a reference substance, Trolox (6-hydroxy-2,5,7,8-tetramethilcroman-2-carboxylic acid) (Merck KGaA, Darmstadt, Germany) was used. Reagents: 0.01M CuCl_2_ (S.C.Chemical Company S.A., Bucuresti, Romania), 7.5 × 10^−3^ M, neocuproine (2,9-Dimethyl-1,10-phenanthroline) (Merck KGaA, Darmstadt, Germany) and acetate buffer. 1mL 0.01M CuCl_2_ solution was mixed with 1 mL neocuproine (7.5 × 10^−3^ M) and 1 mL acetate buffer. At this solution, the 1.1 mL sample (alcoholic extract) was added. For the blank, 50% ethanol was used. The absorption was read after 30 min at 450 nm using a UV–VIS spectrophotometer (Analytic Jena Specord 205, Jena, Germany). All the determinations were performed in triplicate.

### 2.5. In Vitro Antimicrobial Activity

Microbial strains: The reference strains used in this study were represented by Gram-positive cocci (*Staphylococcus aureus* ATCC 25923, *Enterococcus faecalis* ATCC 51299), Gram-negative bacilli (*Salmonella enterica* ATCC 14028, *Shigella flexneri* ATCC 12022, *Escherichia coli* ATCC 25922, *Klebsiella pneumoniae* ATCC700603, *Pseudomonas aeruginosa* ATCC 27853) and yeasts (*Candida albicans* ATCC 10231, *Candida parapsilosis* ATCC 22019).

#### 2.5.1. Disk Diffusion Method

The disk diffusion method was performed according to the CLSI (Clinical Laboratory and Standards Institute Inc.) guidelines [36,37,38,39]. Ten microliters of the OB extract were deposited onto a blank paper disk (BioMaxima, Lublin, Poland) and was then placed on the surface of Mueller-Hinton agar plates (Sanimed, Bucharest, Romania), inoculated with 100 µL of a microbial suspension (0, 5 McFarland) in saline solution. All the testing plates were incubated at 37 °C for 24 h. The reading of the inhibition zones was performed in millimeters. All the disk-diffusion tests were examined in triplicate. For the positive control, we used gentamycin or fluconazole disks (Bio-Rad, Marnes-la-Coquette, France), while for the negative control, we used a disk impregnated with 70% ethanol.

#### 2.5.2. Determination of Minimum Inhibitory Concentration (MIC), Minimum Bactericidal Concentration (MBC) and Minimum Fungicidal Concentration (MFC)

The OB extract was tested by the broth dilution method in accordance with the CLSI and European Committee on Antimicrobial Susceptibility Testing (EUCAST) [36,37,38,39]. A concentration of 200 μg/mL in Mueller Hinton broth (Sanimed, Bucharest, Romania) of the OB extract was initially prepared. Serial twofold dilutions of the OB extract were undertaken in five test tubes, which were inoculated with 5 × 10^5^ microorganisms/mL. After incubating the test tubes at 37 °C for 24 h, the minimum inhibitory concentration (MIC, the lowest concentration that yielded no growth) was observed. All the broths with no visible growth were inoculated on agar + 5% sheep blood or Sabouraud agar (Sanimed, Bucharest, Romania) in order to determine the minimum bactericidal concentration (MBC) or minimum fungicidal concentration (MFC, the lowest concentration which killed 99.9% of the microorganisms). The medium plates were incubated at 37 °C, for 24 h and the MBC or MFC was determined.

### 2.6. Cell Culture

The A375 cell line (ATCC, Manassas, VA, USA) was cultured in Dulbecco’s Modified Eagle’s Medium (DMEM; Gibco BRL, Invitrogen, Carlsbad, CA, USA), supplemented with 10% fetal calf serum (FCS; PromoCell, Heidelberg, Germany) and 1% penicillin/streptomycin mixture (Pen/Strep, 10,000 IU/mL; PromoCell, Heidelberg, Germany), as previously described [40]. Melanoma cells were passaged at confluence after treatment with 5 mM Ethylenediaminetetraacetic acid (EDTA).

### 2.7. Antiproliferative MTT Assay

The growth-inhibitory activity of the extracts and compounds were determined by the standard MTT (3-(4,5-dimethylthiazol-2-yl)-2,5-diphenyltetrazolium bromide) dye uptake method on the A375 human melanoma cell line. Experiments were carried out in the same way, as previously described [33]. Briefly, cells were plated into 96-well plates at a density of 5000 cells/well, and incubated with selected concentrations of the extracts (10, 30, and 60 mg/mL) under cell-culturing conditions. After incubation for 72 h, 5 mg/mL MTT (3-(4,5-dimethylthiazol-2-yl)-2,5-diphenyltetrazolium bromide) solution was added and the samples were incubated for another 4 h. The precipitated form azan crystals were dissolved in dimethyl sulfoxide and the absorbance was measured at 545 nm with a microplate reader. Wells with untreated cells were utilized as the control. The results are presented as the mean of the three different experiments.

### 2.8. Anti-Migratory Activity Evaluation Using the Scratch Assay Method

The scratch assay technique is a wound-healing approach that consists of the generation of a scratch in a cell confluent monolayer and supervises the gap filling by taking pictures for 24 h.

A375 cells were plated at a density of 2 × 10^5^ cells/well in a 12-well plate. When confluence of 90% was reached, the media was discarded and a scratch was made in the middle of each well, followed by the rinsing of cells with phosphate-buffered saline (PBS). After this step, the cells were treated with different concentrations (10, 30, and 60 μg/mL) of the test compound and cell migration was observed by taking pictures at different intervals with an Olympus IX73 inverted microscope documented with a DP74 digital camera (Olympus, Tokyo, Japan). Quantification of A375 cell migration was performed by measuring the cell-free area that resulted after 24 h, using the inverted microscope digital camera software’s measurement function, CellSense Dimension 1.17 (Olympus, Tokyo, Japan).

### 2.9. Detection of Apoptosis via 4′,6-Diamidino-2-Phenylindole (DAPI) Staining

A375 cells were plated to an initial density of 5 × 10^5^ cells/well onto 6-well plates overnight. The following day, the medium was removed and the cells were stimulated with a fresh one containing the test compound to a final concentration of 10, 30 and 60 μg/mL, respectively, for 72 h. At the end of stimulation period, the medium was removed from the wells and the cells were washed with ice-cold PBS twice and were fixed with 4% paraformaldehyde in PBS, permeabilized with 2% Triton-X/PBS for 30 min, followed by a blocking step with 30% FCS/0.01% Triton-X. Finally, the cells were washed with PBS and stained with 4′,6-diamidino-2-phenylindole (DAPI, 300nM) in a dark chamber for 15 min. Fluorescent images were taken at a magnification of 40×, with a fluorescence inverted microscope Olympus IX73, equipped with an integrated DP74 digital camera (Olympus, Tokyo, Japan).

### 2.10. High-Resolution Respirometry Studies

The mitochondrial respiratory function was assessed using high-resolution respirometry (Oroboros Oxygraph-2k Ltd.–Oroboros Instruments, Innsbruck, Austria) at 37 °C. Mitochondrial respiratory rates were attained by following a substrate-uncoupler-inhibitor titration (SUIT) protocol, which was adapted in order to obtain both the separate and conjunctive electron flow from complex I (CI) or/and complex II (CII). For the analysis, 1 × 10^6^/mL cells were suspended in mitochondrial respiration medium (MiR05: 110 mM sucrose, 3 mM MgCl_2_.6H_2_O, 0.5 mM Ethylene glycol-bis(2-aminoethylether)-N,N,N′,N′-tetraacetic acid (EGTA), 60 mM K-lactobionate, 20 mM taurine, 10 mM KH_2_PO_4_, 20 mM 4-(2-Hydroxyethyl)piperazine-1-ethanesulfonic acid (HEPES), 1 g/L Bovine Serum Albumin (BSA), adjusted to pH 7.1 at 37 °C) [41]. Data acquisition and analysis was performed with DatLab software (Oroboros Instruments, Innsbruck, Austria). The specific oxygen flux was expressed as pmol/s per cm^3^ volume.

### 2.11. Experimental Protocol for Permeabilized Cells

A375 human melanoma cells were trypsinized, washed with PBS and resuspended in MiR05 medium at a concentration of 1 × 10^6^ cells/mL. Cell membranes were permeabilized with digitonin (35 μg/10^6^ cells) in order to evaluate the extended functional oxidative phosphorylation (OXPHOS) by allowing for the entry of external added compounds into the cytosol. Digitonin concentrations were previously optimized in our laboratory, following a stepwise digitonin titration, according to a protocol formerly described by Pesta and Gnaiger [41].

Routine respiration was assessed after the cells were suspended in a substrate-free media. We further proceeded with the SUIT protocol, which began with plasma membrane permeabilization by digitonin and then CI substrates: glutamate (G 10 mM) and malate (M 5 mM) were added. The respiration in basal conditions was obtained, namely State 2_CI_. Then, adenosine diphosphate (ADP 5 mM) was added in order to obtain the active respiration dependent on CI, known as OXPHOS_CI_. After the addition of a CII substrate, succinate (S 10 mM), the maximal OXPHOS capacity, determined by both CI and CII, was achieved. Afterwards, an ATP synthase inhibitor, oligomycin (omy 1 μg/mL), was added in order to inhibit ATP synthesis, resulting in a non-phosphorylated state and a return to basal respiration, dependent on both CI and CII (State 4_CI+CII_). The maximal respiratory capacity of the electron transport system (ETS_CI+CII_) was established following successive titrations with a known uncoupler, carbonyl cyanide p-trifluoro-methoxyphenyl hydrazone (FCCP 1 μM/addition). For the evaluation of the electron transport system dependent only on CII (ETS_CII_), a CI inhibitor, rotenone (Rot 0.5 μM), was added. At the end of the experiment a complex III (CIII) inhibitor, antimycin A (Ama 2.5 μM), was added to produce the complete inhibition of the electron transport chain and to determine the residual oxygen consumption (ROX). The mitochondrial respiratory rates were corrected for ROX.

The flux control ratios (FCRs) express various features of mitochondrial respiration that are independent of cell size and mitochondrial content and involve the calculation of the following: OXPHOS_CI+II_/ETS_CI+II_ (P/E), State 2_CI_/ETS_CI+II_ (L/E), Routine/ETS_CI+II_ (R/E) and OXPHOS_CI+II_/State 4_CI+II_ (RCR).

The P/E ratio represents the function of the phosphorylation system, the L/E ratio evaluates the potential of mitochondrial coupling, the R/E ratio indicates how close the routine respiration is to the maximal ETS capacity and RCR (respiratory control ratio) increases in fully coupled mitochondria [41].

### 2.12. Chick Chorioallantoic Membrane (CAM) Assay

Fertilized hen (*Gallus gallus domesticus*) eggs were used to assess the changes induced by OB extracts in the angiogenesis and tumor angiogenesis process. Fertilized eggs were incubated at 37 °C and 50% humidity until the third embryonic day of development (EDD) when 5–6 mL of albumen was removed and a window was cut on the upper side of the eggs [42,43].

The influence on the angiogenesis process was evaluated on normal developing chorioallantoic membranes (CAMs) from the EDD 7, when 10 µL of each concentration of OB extract in the concentration of 60 µg/mL were applied inside plastic rings, which were disposed on each developing CAM. To assess the tumor angiogenesis, the same basic protocol was used, with the inoculation of A375 melanoma cells 10^5^ cells/5 µL on EDD 10, and subsequently the same OB extract was pipetted inside the plastic rings.

Samples were applied daily for three days (24 h, 48 h and 72 h) and relevant photographs were captured in ovo by means of a stereomicroscope (SteREO Discovery.V8, Zeiss, Göttingen, Germany), coupled to a digital camera (Axio CAM 105 color, Zeiss, Göttingen, Germany) and further processed by AxioVision software (SE64. Rel. 4.9.1, Zeiss, Göttingen, Germany), ImageJ (Version 1.50e https://imagej.nih.gov/ij/index.html) and GIMP (v2.8 GNU Image Manipulation Program https://www.gimp.org/).

The investigations were performed by comparing the effects of the tested OB extract to dimethyl sulfoxide (DMSO) as solvent control (0.1% DMSO *v*/*v* in double distilled water). All the experiments were performed in triplicate.

### 2.13. In Vivo TPA-Induced Ear Inflammation Protocol

The evaluation of the anti-inflammatory potential of the hydroalcoholic extract of the aerial part of the OB was conducted, as previously described, using the 12-o-tetradecanoylphorbol−13-acetate (TPA)-induced ear inflammation protocol on the SKH1 female mice strain-Charles River Laboratory (Budapest, Hungary). The European Directive 2010/63/EU and the national law 43/2014 were respected. The animals were euthanized following the guidelines of the American Veterinary Medical Association (AVMA) for the Euthanasia of Animals (2013 Edition). The experiment followed the rules and was approved by the Bioethical Committee of the Faculty of Pharmacy of Victor Babes University of Medicine and Pharmacy Timisoara, Romania (No. 1/13.01.2020) [44,45]. Five groups (n = 5), each consisting of 7 mice, were included in the study as follows: (i) control (surface ~1 cm^2^-inner surface of the left ear); (ii) animals, to which the experimental animal model of inflammation was induced by using TPA dissolved in acetone (20 µL/mouse ear) (surface ~1 cm^2^-inner surface of the right ear); (iii) animals, to which acetone (20 µL/mouse ear) was applied on the ear (surface ~1 cm^2^-inner surface of the right ear); (iv) animals, to which indomethacin cream (4%) was typically applied as the treatment after the induction of the experimental animal model of inflammation (surface ~1 cm^2^-inner surface of the right ear); (v) animals to which OB extract (300 µg/cm^2^) was typically applied as the treatment after the induction of the experimental animal model of inflammation (surface ~1 cm^2^-inner surface of the right ear) [46].

### 2.14. Histopathological Assessment of Mice Ears

An assessment of the ear skin of the mice was performed at the end of the experiment (6 h post inoculation) when the mice were sacrificed for histopathological evaluation. The mice used in this experimental research were euthanized under anesthesia, followed by cervical dislocation, which are techniques that are in agreement with the AVMA Guidelines for Euthanasia. The control and treated mice were necropsied, and their ears were harvested. After sampling, the fresh specimens were immediately immersed in a fixative that maintains cellular integrity. To give perfectly adequate tissue preservation, 10% neutral phosphate-buffered formalin (pH 7) was used. Samples were processed with Leica TP1020 Semi-enclosed Benchtop Tissue Processor (Leica Biosystems Nussloch GmbH, Nussloch, Germany) and embedded in paraffin wax with HistoCore Arcadia H+C Embedding System (Leica Biosystems Nussloch GmbH, Nussloch, Germany). After that, 2.5 µm-thick sections were cut using a Leica Biosystems RM2245 Semi-Automated Rotary Microtome (Leica Biosystems Nussloch GmbH, Nussloch, Germany) and spread, stretched on glass slides, deparaffinized in toluene and rehydrated with ethanol in decreasing concentrations. Next, the tissue sections were stained with conventional hematoxylin and eosin (HE), dehydrated with ethanol in increasing concentrations and coverslipped with Entellan^®^ New. The slides were examined by the pathologist using a binocular microscope Leica DM750 LEICA (Leica Microsystems AG, Heerbrugg, Switzerland). Image acquisition and analysis were performed using a Leica ICC50 W Camera (Leica Microsystems AG, Heerbrugg, Switzerland).

### 2.15. Statistics

Statistics were conducted, as previously described [43]. For data collection and presentation, the Prism software package (Graph Pad Prism 5.0 for Windows, https://www.graphpad.com/scientific-software/prism/) was employed. Data are presented as mean ± SD (standard deviation). Each experiment was repeated at least three times. (* *p* < 0.05; ** *p* < 0.01; *** *p* < 0.001; **** *p* < 0.0001 vs. control, calculated by one-way analysis of variance (ANOVA), followed by a Tukey post-test).

## 3. Results

**Figure 1:**
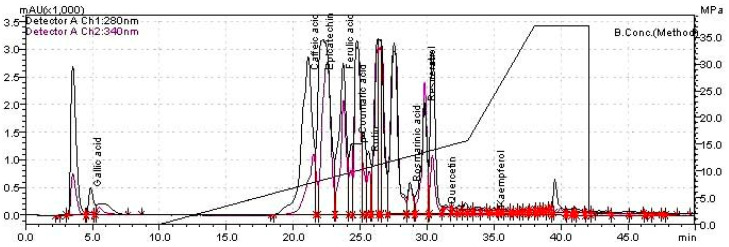
Liquid chromatogram of the *Oenothera biennis* L. (OB) extract. (chromatographic conditions: 280 nm and 340 nm, column EC 150/2 NUCLEODUR C18 Gravity SB 150 × 2 mm × 5 μm, mobile phases A: water acidified with formic acid at pH-3, B: acetonitrile acidified with formic acid at pH 3. Gradient chromatographic separation program: 0.01–20 min 5% B, 20.01–50 min 5–40% B, 5–55 min 40–95% B, and 55–60 min 95% B. The solvent flow rate was 0.2 mL/min at 20 °C, and compounds identification was done using external standards).

The liquid chromatogram (LC) of individual polyphenols is presented in Figure 1. Among the screened phytochemicals, the following compounds were detected: gallic acid (1.064 mg/g), caffeic acid (11.525 mg/g), epicatechine (78.40 mg/g), coumaric acid (0.26 mg/g), ferulic acid (1.250 mg/g), rutin (3.528 mg/g), and rosmarinic acid (1.601 mg/g).

The ethanolic extracts obtained from the aerial part of the OB have shown an amount of total phenolic content of 631.496 µgGAE/mL extract. As shown by the consecrated CUPRAC assay, the elicited antioxidant activity was 7258.67 μmolTrolox/g extract. The results are displayed in Table 1.

The MIC values for *Candida* spp. (50 μg/mL) and *S. aureus* (100 μg/mL) were lower than those for *E. faecalis* (200 μg/mL) or Gram-negative bacilli. In addition, the values of MBC were higher than those of MIC. Based on these results, we can declare that the tested extract may have a bacteriostatic effect against the bacterial strains tested, but it is only bactericidal against *S. aureus* and fungicidal and against all strains of the *Candida* tested (Table 2).

**Figure 2:**
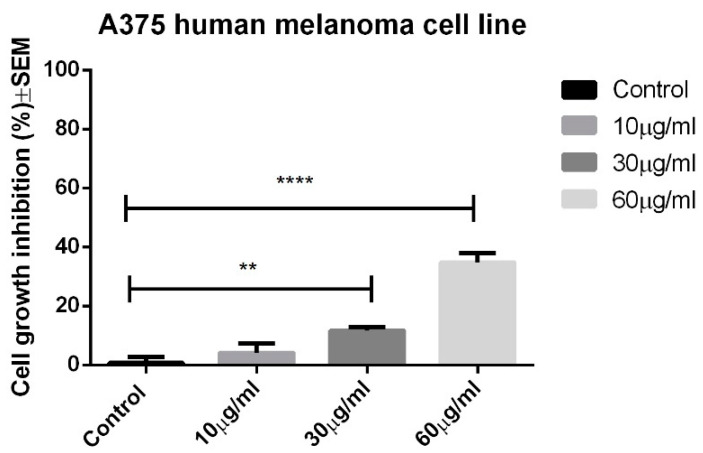
Antiproliferative potential of the OB extract against the A375 human melanoma cell line. One-way analysis of variance (ANOVA) was employed to determine the statistical differences, followed by a Tukey post-test (** *p* < 0.01; **** *p* < 0.0001).

In the experimental conditions, the OB extract only manifested significant antiproliferative activity against the A375 human melanoma cell line at the highest tested concentration, namely 60 μg/mL, leading to an average cell growth inhibition of 34.82 ± 2.721. The results can be seen in Figure 2.

To assess the effect of the OB extract on human melanoma (A375) cell migration and proliferation potential, a wound-healing test was employed.

**Figure 3:**
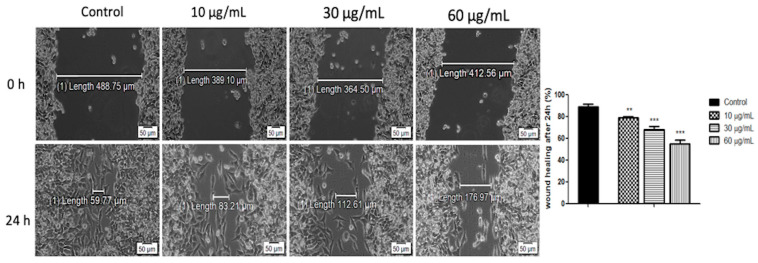
OB extract (10, 30, and 60 μg/mL) activity on A375 cell migration and proliferation potential. Progression of cell migration was monitored by imaging the wound healing initially and 24 h post-stimulation. Data are calculated as the percentage of the scratched surface at 24 h, and were compared to the same scratched surface at 0 h. Scale bars denote 50 μm. The results represent the mean values ± standard deviation (SD) of three independent experiments. One-way ANOVA was employed to determine the statistical differences, followed by a Tukey post-test (** *p* < 0.01; *** *p* < 0.001).

As shown in Figure 3, the migration potential of A375 cells was hampered by the OB extract in a concentration-dependent manner. The lowest concentration (10 μg/mL) induced a wound closure of 79.05% ± 1.02, which translates to a 20.95% inhibition rate. Concentrations of 30 and 60 μg/mL manifested a more significant repression on the proliferation of human melanoma–A375 cell, displaying wound closure rates of 68.20% ± 2.98 and 55.32% ± 3.09, respectively, after 24 h.

**Figure 4:**
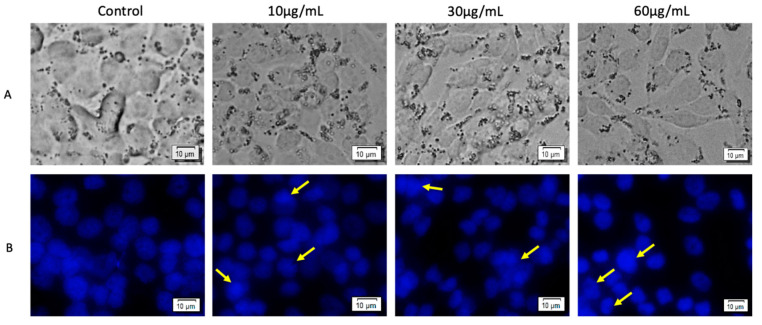
A375 cells treated with the OB extract at different concentrations (10, 30, and 60 μg/mL), for 72 h. (**A**) Bright field microscopy was employed to analyze cell morphology; (**B**) 4′,6-diamidino-2-phenylindole (DAPI) staining was performed for apoptotic morphological characteristics (DNA condensation and nuclear membrane blebbing are indicated by yellow arrows).

Human melanoma (A375) cells treated with the OB extract at concentrations of 10, 30, and 60 μg/mL showed a slight decrease in cell density in a concentration-dependent manner, when analyzed by bright field microscopy (Figure 4A). Staining of A375 cell culture with DAPI displayed specific signs of apoptosis: DNA condensation and nuclear membrane blebbing (indicated by yellow arrows), in comparison to control cells, had a chromatin density that was equally dispersed (Figure 4B). However, the highest concentration (60 μg/mL) increased the incidence of apoptotic markers.

**Figure 5:**
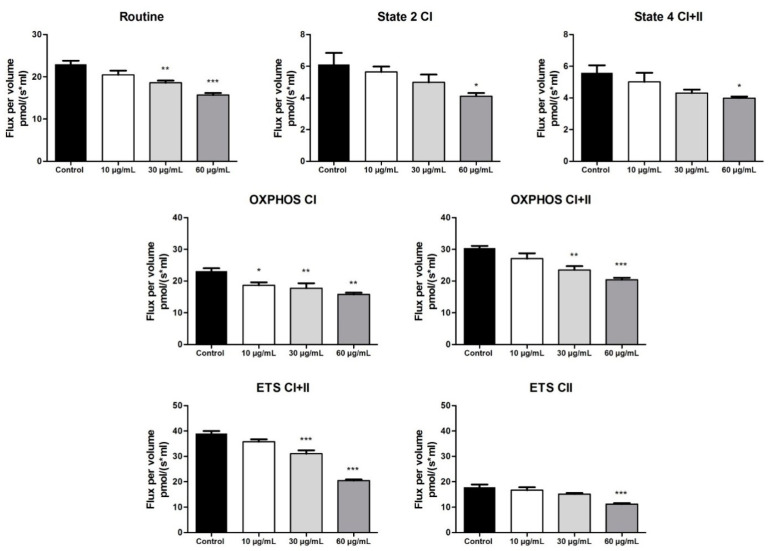
OB extract (10, 30, 60 µg/mL) effects on A375 human melanoma cells on mitochondrial respiratory rates at 72 h post-stimulation. Data are expressed as mean ± SD of four independent experiments. (* *p* < 0.05; ** *p* < 0.01; *** *p* < 0.001 vs. control calculated by one-way ANOVA, followed by a Tukey post-test). (CI: complex I; CII: complex 2; OXPHOS: oxidative phosphorylation; ETS: electron transfer system).

**Figure 6:**
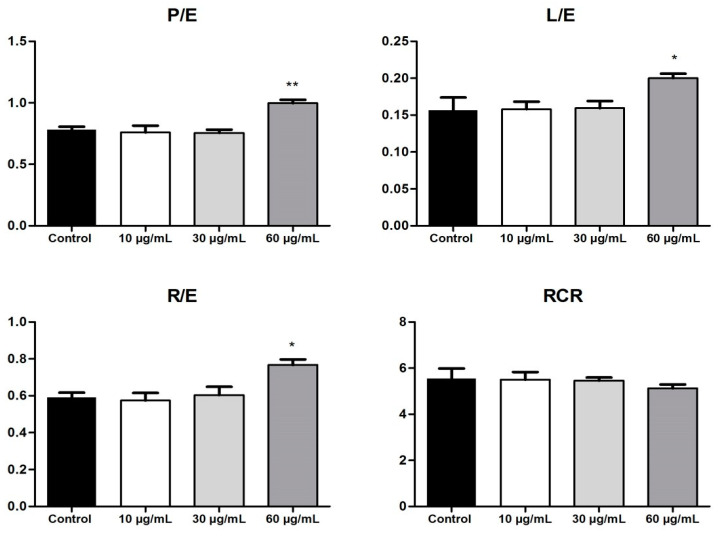
OB extract (10, 30, and 60 µg/mL) effects on the flux control ratios (FCRs). Data are expressed as mean ± SD. (* *p* < 0.05; ** *p* < 0.01; vs. control, calculated by one-way ANOVA, followed by a Tukey post-test). The FCRs involve the calculation of the following: (P/E) OXPHOS_CI+II_/ETS_CI+II_; (L/E) State 2_CI_/ETS_CI+II_; (R/E) Routine/ETS_CI+II_; and (RCR—respiratory control ratio) OXPHOS_CI+II_/State 4_CI+II_. P/E—oxidative phosphorylation _CI+II_/electron transfer system _CI+II_; L/E—State 2_CI_/electron transfer system _CI+II_; R/E—routine/electron transfer system _CI+II_.

The effect of the OB extract was further evaluated for mitochondrial respiratory function by means of high-resolution respirometry (Figure 5 and Figure 6). At 72 h post-stimulation, the OB extract elicited a dose dependent decrease in all mitochondrial respiratory rates, as compared to the control (untreated cells) (Figure 5). A reduction in State 2_CI_ and State 4_CI+II_ is an indicator that the OB extract, especially at the highest dose tested (60 µg/mL), provoked a decrease in the oxygen consumption when the phosphorylation system is in an inactivated state. Furthermore, the reduction of OXPHOS_CI_ and OXPHOS_CI+II_, together with a significant decrease in ETS_CI+II_ and ETS_CII_, indicates an inhibitory effect in the active respiration and a reduction in the maximal respiratory capacity.

The FCRs that express different features of the mitochondrial respiration are presented in Figure 6. The OB extract, at the highest dose tested, significantly increased the FCRs (P/E; L/E; R/E) at 72 h post-stimulation. An elevation in the P/E ratio indicates that the extract modifies the phosphorylation system and thus can limit the OXPHOS capacity. Another feature that indicates a limitation in the OXPHOS system is related to the increase in the R/E ratio (OB extract 60 µg/mL vs. control). Stimulation with the OB extract increased the L/E ratio and determined a slight reduction in the RCR, which are effects that can be correlated with mitochondrial uncoupling and can lead to mitochondrial dysfunction. Altogether, the data obtained shows that the OB extract induced a dose-dependent impairment of the mitochondrial function.

Considering the relevant effect of the OB extracts on the viability, apoptotic process and mitochondrial function of A375 melanoma cells at the highest tested concentration of 60 μg/mL, we decided to investigate in ovo the angiogenic implications of the OB extract at this particular concentration.

**Figure 7:**
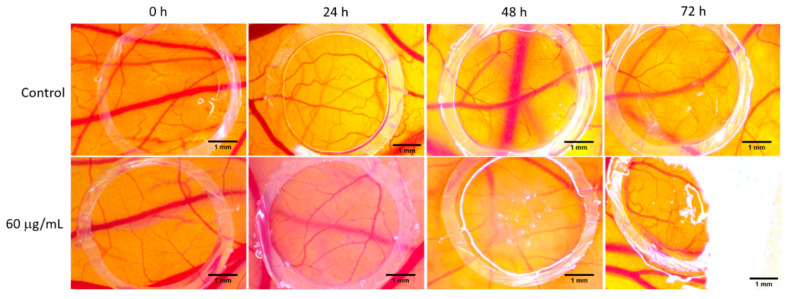
Normal angiogenesis evaluation in chorioallantoic membrane (CAM) assay. The OB extract (60 μg/mL) was applied next to the dimethyl sulfoxide (DMSO) as the control; stereomicroscope photographs were recorded at 0 h, 24 h, 48 h and 72 h. CAM, chorioallantoic membrane; OB, *Oenothera biennis*; DMSO, dimethyl sulfoxide.

The normal process of angiogenesis was affected by the OB extract. Signs of vessel impairment could only be observed after 48 h and two applications, and were mainly observed as the reduction in the capillary caliber, which is a lower number of honeycomb-like interconnections and is a characteristic for this developing interval (Figure 7). The progressive effect was even perceived as stronger at 72 h, inducing a discreet, irregular aspect of the vessel’s architecture (Figure 7). DMSO, in a concentration of 0.1%, used as solvent, did not affect the vessel’s development, showing a normal pattern of high-density capillaries. The extract was well tolerated, registering high survival rates of the embryos.

**Figure 8:**
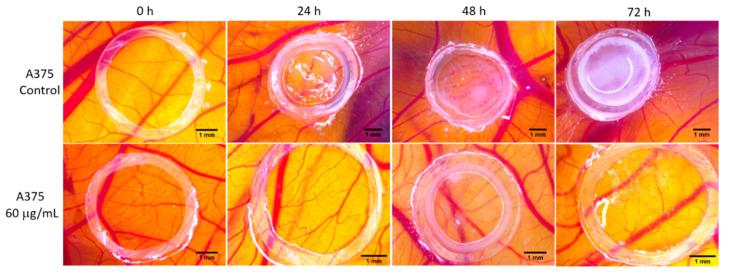
Tumor angiogenesis evaluation in the CAM assay using A375 melanoma cells. In ovo tumor cells were exposed to the OB extract (60 μg/mL), next to DMSO as the control; stereomicroscope photographs were recorded at 0 h, 24 h, 48 h and 72 h. CAM, chorioallantoic membrane; OB, *Oenothera biennis*; DMSO, dimethyl sulfoxide.

After inoculating A375 melanoma cells onto the developing CAMs, we evaluated the tumor cell distribution and the resulting vascular reaction after exposure to the OB extracts. Some observations could be made 24 h after inoculation, but an important modification was noted 48 and 72 h post inoculation. Tumor cells formed semi-compact areas when treated with DMSO, with characteristic spoke-wheel vascular patterns, and when treated with OB60, a limited cell distribution, moderate angiogenic reaction with some irregularities and a lower number of newly formed capillaries were observed (Figure 8).

The angiogenic reaction was not totally inhibited, but no spoke-wheel patterns were observed. Nevertheless, the number of fine capillaries was reduced in comparison to the control samples, and they had disrupted aspect and trajectory. A high survival rate of the embryos was noted for all tumor inoculated CAMs when exposed to the test samples, indicating good tolerability of the OB extract on this melanoma model.

**Figure 9:**
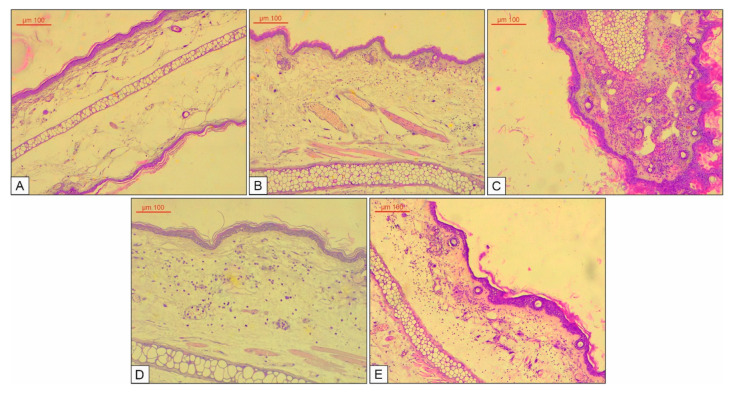
Histological aspects of the mice’s ear skin, hematoxylin and eosin (HE) staining: (**A**) Control group–with no intervention, magnification ×10; (**B**) acetone group—edema, discrete interstitial inflammatory infiltrate, magnification ×20; (**C**) 12-o-tetradecanoylphorbol−13-acetate (TPA) group—marked dermo-epidermal and interstitial inflammatory infiltrate, magnification ×20; (**D**) TPA + indomethacin group—mild inflammation, magnification ×20; (**E**) topical application of the OB extract, moderate interstitial inflammation magnification ×20.

Mice ear specimens from the control group had a normal histological structure (Figure 9A). In the acetone-treated group, interstitial edema, congestion of the blood vessels and a discrete interstitial inflammatory infiltrate can be observed (consisting of neutrophil polymorphonuclear leukocytes) (Figure 9B). The application of TPA induced notable inflammatory changes on mice ears: the appearance of a marked spot/focal dermo-epidermal and interstitial inflammatory infiltrate (consisting of neutrophil polymorphonuclear leukocytes); in the blood vessels, the endothelium is turgescent, and the appearance of leukocytes margination can be observed; the epidermis is thickened with reactive cell modifications (Figure 9C). The subsequent application of indomethacin after initial treatment with TPA (Figure 9D) induced a significant reduction in inflammatory processes, as compared to the TPA group. The inflammatory infiltrate was composed of a few neutrophils that were diffusely disposed in the interstitial space; the interstitial edema was mild; congestion and leucocytes margination of the blood vessels were reduced. Subsequent application of the OB extract (Figure 9E) after initial treatment with TPA showed an anti-inflammatory effect. Interstitial edema and congestion of the blood vessels were mild. Moreover, the inflammatory infiltrate was moderate, with a diffuse distribution of neutrophil polymorphonuclear leukocytes in the dermis and interstitial space. However, the epidermis was focally enlarged, as compared to the control.

## 4. Discussion

Previous studies revealed that the OB extracts contain polyphenolic compounds, which is a potential source of antioxidants [47,48,49]. However, the results regarding the polyphenolic fractions of the OB herb are few, and previous studies focused specifically on the polyphenolic composition of seed.

Ratz-Łyko et al. (2014) reported that OB seed extracts contained catechin, quercetin, gallic acid, and ferulic acid, while Zadernowski et al. (2002) highlighted that in OB seed extracts, there was the presence of flavonoids and hydroxicinamic acids (gallic, ferulic, syringic, protocatechuic, and p-hydroxybenzoic acids) at a concentration of 125 mg/kg of the total seed amount, including approximately 85 mg/kg in the free form, 27 mg/kg in the ester form, and 11 mg/kg in the glycoside form [47,50]. Regarding the individual polyphenols content, Karamac et al. reported a gallic acid range of 5 to 309 mg/g in some plant extracts like *Camellia sinensis* L., *Arctostaphylos uva-ursi* L., *Corylus avellana* L., *Oenothera biennis* L., and *Vitis vinifera* L [51].

The comprehensive review of Timoszuk et al. presents data about the phytochemical composition of different vegetable products (aerial parts, leafs, root, seeds, and oil) obtained from OB. Regarding the aerial part of the plant, the review describes the phytochemical composition of methanolic extract and assigns the main phytochemicals to the class of phenolic acids and flavonoids. From the class of phenolic acids, gallic and ellagic acids, together with their ester derivatives, as well as caffeic acid, 3-p-feruloylquinic acid, 4-p-feruloylquinic acid, and 3-p-coumaroylquinic acid andvaloneic acid, have been identified [5]. Similar phytochemical fingerprints regarding the phenolic acids content was obtained within this study in the case of ethanolic extracts. As described above, in the set experimental conditions of this study: gallic acid, caffeic acid, epicatechine, coumaric acid, ferulic acid and rosmarinic acid have been detected as the main polyphenols. Regarding the flavonoid profile, Timoszuk et al. identified different glycosides of myricetin, quercetin and kaempferol [5]. Within this study, rutin was found to be the predominant flavonoid. Granica et al. also investigated the chemical composition of extracts prepared from aerial parts of evening primrose. Their conclusions were in agreement with those of other research groups, namely that extracts are rich in polyphenols. They identified 39 phytocompounds. Moreover, they also outlined the radical scavenging potential of the *O. biennis* L. aerial part extracts [10]. The complete review by Munir et al. provides updates concerning the pronounced antioxidant activity of ethanolic extracts obtained from OB [26].

According to previous studies, OB exhibited pronounced antioxidant activity [52,53,54]. Peschel et al. (2007) analyzed the seed extract of OB and reported a total phenolic content of 495.38 mg GAE/g. Similar results (466.67 mg GAE/g) were obtained by Ratz-Łyko et al. (2014) for seedcake extracts of OB [50,53]. The TP values obtained for the ethanolic extract of OB described within this study are slightly higher, as compared to those reported in other *Onagraceae* species: *O*. *paradoxa* (469 to 388 mg GAE/g [55] or *O. rosea* ethanolic extract 135.68 ± 8.20 mg GAE/g [56]. The differences may be due to the geographical and climatic conditions, the parts of the plants analyzed, and/or the extraction methods.

The literature presents some reports regarding the antibacterial effect of plant products obtained from evening primrose. Methanolic extracts of seeds of OB were tested using the disk diffusion assay against *S. aureus, P. aeruginosa, E. coli* and *C. albicans*. The extracts elicited significant antibacterial effects against all the four tested strains [15]. Additionally, methanolic and aqueous extracts obtained from *O. rosea* L’Hér. ex Aiton, commonly known as pink evening primrose, were assigned with an antibacterial effect for pathogens existing within the sphere of the digestive tract [26]. Hamedi and Vatani evaluated the antibacterial and antifungal effect of the oil obtained from the seeds of this species against both bacteria (*S. aureus*, *S. epidermidis*, and *P. aeruginosa)* as well as fungus *(C. albicans* and *A niger)* in a dose range of (10–1000 mg/L). The results have shown, on the one hand significant inhibition of the growth of bacteria, and on the other hand, the stimulation of the growth of fungus [57]. Evening primrose seedcake extracts, before and after hydrolyses, elicited a strong antibacterial activity against *P. aeruginosa* [50]. In a recent study by Singh et al., the antimicrobial and antiproliferative potential of the compounds, isolated from the roots of this plant, was evaluated. The study concluded that the screened phytocompounds present moderate antimicrobial activity, oenotheralanosterol B and dihydroxyprenylxanthone acetylated, which is the most active [13]. In a comprehensive in vitro and in vivo study by Park et al., they demonstrated the efficiency of OB ethanolic extract against *Salmonella typhimurium* [58].

The in vitro results obtained in the present study sustained the hypothesis that all the test concentrations (10, 30, and 60 μg/mL) of the OB ethanol extract manifested anti-melanoma potential in a dose-dependent manner. The data were augmented by all the in vitro assessments performed in this study: MTT test, scratch assay and DAPI staining. However, the most significant anti-melanoma effect was observed after exposure to the human melanoma—A375 cells had the highest concentration (60 μg/mL) of OB extract and revealed an inhibition rate of cell growth of 34.82 ± 2.721, and a migration rate of almost half (55.32% ± 3.09), as compared to that of control cells, and multiple hallmarks of apoptosis. These results are corroborated with those presented by Jaszewska and collaborators who reported the cytotoxic effect of different extracts of *O. paradoxa* on human skin melanoma HTB-140 cells. The mechanism implicated in the cytotoxic effect revealed high ROS (reactive oxygen species) production and a low level of glutathione, without caspase-3 activation [29]. Moreover, the defatted seed extract of *O. paradoxa* Hudziok, rich in pentagalloyloglucose and procyanidins, significantly augmented the sensibility of melanoma cells to the action of the drug vincristine [26].

Nevertheless, a possible benefit in the recovery of cancerous skin lesions for evening primrose oil (EPO) was discussed in the study by Ramesh et al. They showed that, in an animal model of two stage skin carcinogenesis, EPO significantly inhibited the formation of papilloma in the promotion stage of the model, and was even assigned to the inhibition of the binding of benzo(a)-pyrene to the DNA of skin cells and to an increased lipid peroxidation process [59]. An essential component of evening primrose oil is gamma-linolenic acid, which was reported to inhibit the growth of B16 melanoma cells [60]. Trying to find natural bleaching agents, Koo et al. demonstrated that saponified evening primrose oil can dose-dependently decrease melanin production, in the case of B16 melanoma cells, via a mechanism that involves a reduction in the activity of enzymes, as well as a decrease in mRNA and protein levels of tyrosinase [61]. Additionally, Kim et al. showed the significant anti-melanogenic potential of *O. laciniata* methanol extract on melan-a cells, referring to the same mechanism that suppresses tyrosinase activity, tyrosinase-related proteins (TRP-1 and TRP-2) and microphthalmia-associated transcription factor-M mARN expression [30].

The scientific literature is scarce regarding the effect of the OB extract at the mitochondrial level. The work designed by Arimura et al. describes the effect of the evening primrose seeds extract (EPE) on mitochondrial membrane potential and the release of cytochrome c by EPE. Following previous analyses, it was indicated that 200 µg/mL EPE significantly decreased mitochondrial membrane potential of the Ehrlich ascites tumor cells, as compared to the non-treated cells. Furthermore, EPE-treated tumor cells showed a dose- and time-dependent release of cytochrome c. The release of cytochrome c precedes the activation of caspase-3, both being characteristic of apoptosis, but this activation was not observed in this study, and thus, the results showed that the activation of caspase-3 cannot be directly involved in EPE-induced apoptosis [62]. In another study, Arimura et al. described that the Ehrlich ascites tumor cells exposed to 200 µg/mL EPE showed a time-dependent translocation of apoptosis-inducing factor to nuclei and that increased levels of mitochondrial Bax could be noticed concomitantly with a loss of cytosolic Bax. Additionally, the group demonstrated that the extract from OB decreased tumor cell viability, an effect that was not reversed with the addition of a specific caspase-3-like protease inhibitor, acetyl-Asp-Glu-Val-Asp-aldehyde (DEVD-CHO) [63].

Arimura et al. further analyzed the OB extract on Ehrlich ascites tumor cells and showed that it provoked an inhibition in DNA synthesis and elicited a dose-dependent accumulation of cells in the G1 phase. Moreover, the study showed that the extract decreased hyperphosphorylated Rb (pRb) levels and increased hypophosphorylated (Rb) levels in a dose and time-dependent manner. Finally, it was indicated that these effects were not suppressed by the addition of catalase [64]. In another paper, Dalla Pellegrina et al. investigated the mechanisms of cell killing induced by gallic acid-containing phenolic fraction from *O. biennis* on leukemia cells using specific inhibitors of different caspases. Inhibitors of caspases 1 and 8 induced an inhibition of the cytotoxic activity of phenolic fraction 4 from *O. biennis*, whereas the effect was not maintained for the inhibitors of caspase 3 and 6. The group indicated that this phenolic fraction 4, obtained from evening primrose seeds, induced apoptosis in leukemia cells through a caspase-dependent pathway [65]. To the best of our knowledge, this is the first report on the effects of the OB extract on mitochondrial respiration.

There are not many studies concerning the possible implication of different evening primrose extracts in the angiogenic process. Evening primrose oil and its principal phytocompound, gamma linolenic acid, were shown to influence chronic inflammatory processes as in rheumatoid arthritis by reducing the expression of proinflammatory and angiogenic cytokines—vascular endothelial growth factor (VEGF) and angiopoietin-1 (ANG-1), and enhanced the anti-inflammatory and anti-angiogenic activities of aspirin and celecoxib [5,66]. Other studies conducted on defatted seeds of evening primrose (*O. paradoxa*), rich in proanthocyanidins, indicate the reduction of breast tumor invasiveness and anti-angiogenic activity by the decreased expression levels of angiogenic VEGF and through the reduction of matrix metallopeptidase 9 (MMP-9) activity [67], and having anti-metastatic effects on colorectal cancer [68]. To the best of our knowledge, we contribute to these findings, by firstly reporting, in ovo, the effects of the OB ethanolic extract on the impairment of vessel development and melanoma formation and on the chick chorioallantoic membrane.

In a comprehensive study that evaluated the anti-inflammatory potential of extracts obtained from the aerial parts of two *Oenothera* species, namely *O. biennis* L. and *O. paradoxa* Hudziok, Granica et al. concluded that both extracts elicit anti-inflammatory activity in a concentration dependent manner by a mechanism that involves the inhibition of hyaluronidase and lipoxygenase [10]. However, an increased number of studies have reported the anti-inflammatory potential of evening primrose oil, which is a property assigned to the main phytochemicals present in the oil, namely linoleic acid and γ-linolenic acid. This property is part of the multi-target effect of this phytochemicals, including inhibition of 5-LOX (5-lipoxygenase), suppression of inflammation mediators such as cytokines (e.g., tumor necrosis factor α (TNF-α)) and interleukins (IL-1β, IL6), and activation of biochemical pathways that lead to the synthesis of series 1 prostaglandins [5]. Moreover, it was observed that the intake of evening primrose supplements can prevent an increased number of inflammatory diseases, including multiple sclerosis [69]. This oil, rich in gamma linolenic acid, has been shown to have synergistic activity with celecoxib in an experimental animal model of rheumatoid arthritis by inhibiting pathological angiogenesis, inflammation, and oxidative stress [66]. The methanolic extract from another species of the *Oenothera* genus, namely *O. rosea* L’Hér. ex Aiton, has been shown to have important anti-inflammatory effects in an animal model of inflammation-type carrageenan-induced rat paw edema. The effect was later demonstrated for the aqueous extract [26]. These findings corroborated with the data presented in this study and shows the anti-inflammatory potential of the ethanolic extract obtained from the OB collected from the southern part of Tunisia (Djerba).

## 5. Conclusions

The molecular fingerprint, in terms of individual polyphenols and flavonoids of the hydroalcoholic extract, were obtained from the aerial part of the OB collected from the southern part of Tunisia (Djerba), and includes: gallic acid, caffeic acid, epicatechine, coumaric acid, ferulic acid, rutin and rosmarinic acid. Total phenolic content was 631.496 µgGAE/mL extract, and the antioxidant activity 7258.67 μmolTrolox/g extract. The tested extract had a mild bacteriostatic effect on the tested bacterial strains. It was only bactericidal against *S. aureus,* but fungicidal against all the *Candida*-tested strains. In the set experimental conditions, the OB extract only manifested significant antiproliferative and pro-apoptotic activity against the A375 human melanoma cell line at the highest tested concentration, namely 60 μg/mL. The migration potential of A375 cells was hampered by the OB extract in a concentration-dependent manner. The OB extract provoked a dose-dependent impairment of the mitochondrial function. Additionally, both normal and tumor angiogenesis were affected by the OB extract at a concentration of 60 μg/mL, and A375 cells did not form compact tumors in ovo. Moreover, the OB extract elicited an anti-inflammatory effect on the experimental animal model of ear inflammation. All these data support the idea that the hydroalcoholic extract obtained from the OB elicit important biological activities in vitro, in ovo and in vivo, constituting a premise for the design of comprehensive experimental animal model studies and then, maybe, even clinical trials in order to fully benefit from the potential of the bioactive molecules present within this extract. To the best of our knowledge, this is the first comprehensive biological evaluation of the hydroalcoholic extract obtained from the aerial part of OB.

## Figures and Tables

**Table 1:** Total phenolic content (TP) and total antioxidant activity (TAC) of the OB extract.

Sample	TP (µgGAE/mL Extract)	CUPRAC (μmol Trolox/g Extract)
OB	631.496 ± 6.381	7258.67 ± 25.152

Legend: CUPRAC—cupric reducing antioxidant capacity.

**Table 2:** Antimicrobial activity of the OB extract.

Bacterial Species	Inhibition Diameters (mm)	MIC (μg/mL)	MBC/MFC (μg/mL)
*K. pneumoniae*	7 mm	200	-
*S. flexneri*	7 mm	200	-
*S. enterica*	7 mm	200	-
*E. coli*	7 mm	200	-
*P. aeruginosa*	7 mm	200	-
*S. aureus*	8 mm	100	200
*E. faecalis*	7 mm	200	-
*C. albicans*	10 mm	50	100
*C. parapsilosis*	10 mm	50	100

Legend: MIC—minimum inhibitory concentration, MBC—minimum bactericidal concentration, and MFC—minimum fungicidal concentration.
